# Cytochrome P450 and P-gp Mediated Herb-Drug Interactions and Molecular Docking Studies of Garcinol

**DOI:** 10.3390/membranes11120992

**Published:** 2021-12-19

**Authors:** Lavanya Bolla, Pratima Srivastava, Velayutham Ravichandiran, Satheesh Kumar Nanjappan

**Affiliations:** 1 Aragen Life Sciences Pvt. Ltd. (Formerly known as GVK Biosciences Pvt. Ltd.), IDA Nacharam, Hyderabad 500076, India; lavanyabolla225@gmail.com (L.B.); pratima.srivastava@gvkbio.com (P.S.); 2 Department of Natural Products, National Institute of Pharmaceutical Education and Research (NIPER), Kolkata, Chunilal Bhawan 168, Maniktala Main Road, Kolkata 700054, India

**Keywords:** garcinol, herb-drug interaction, CYP inhibition, molecular docking, P-gp

## Abstract

Garcinol is an active constituent of *Garcinia indica* and *Garcinia cambogia*. Recent studies have proven that garcinol has anti-inflammatory, anti-cancer, and anti-oxidant activities. The objective of this study was to evaluate the inhibitory effects of garcinol on the activities of the drug metabolizing cytochrome P450 (CYP) isozymes to predict potential herb-drug interactions with co-administered drugs. Garcinol was incubated with a mixture of rat liver microsomes and eight CYP probe substrate cocktail under optimized incubation conditions and the samples were analyzed using a validated method on LC-MS/MS. Garcinol showed strong inhibition with IC_50_ values of CYP1A2 (7.6 µM), CYP2C9 (8.0 µM), CYP2B6 (2.1 µM), CYP2D6 (9.5 µM), and CYP3A4 (5.1 µM), respectively, and moderate inhibition towards CYP2C19 (16.4 µM) and CYP2E1 (19.0 µM). Molecular docking studies were performed on garcinol against the active sites of CYP2B6 and CYP3A4 proteins. These results further confirmed that the inhibitory activity of garcinol occurred by occupying the active sites of these human CYPs and by making favorable interactions with its key residues. In-vivo CYP inhibition studies were carried out in Sprague-Dawley rats. These results suggest garcinol may cause herb-drug interactions, mediated by inhibition of CYPs involved in drug metabolism in-vivo by altering the pharmacokinetic parameters like AUC and C_max_ in a clinically significant manner. Garcinol was found to upregulate the expression and activity of P-gp in western blotting study and P-gp inhibition study in-vivo. These findings give a clear understanding to predict potential herb-drug/drug-drug interactions of garcinol for safe clinical use in future.

## 1. Introduction

*Garcinia cambogia* (Malabar tamarind) and *Garcinia indica* (kokum) are small tropical trees grown in Africa, Asia (western ghats of India), and Pacific regions [1]. Parts or extracts of these food plants have been used in traditional Ayurvedic medical system to treat various gastric ailments and skin-related problems because of its therapeutic efficiency [2]. The ripe fruits and dried fruit rinds are used as condiment and flavoring agent without any adverse effect. Many natural products have been extracted from various parts of these trees namely hydroxycitric acid (HCA), hydroxycitricacid lactone, citric acid, oxalic acid, malic acid, ascorbic acid, polyphenols, anthocyanin, garcinol, camboginol, isogarcinol, guttiferones, and xanthochymol [3,4]. An α, β-dihydroxy tricarboxylic acid, HCA is one of the main ingredients of the fruit rind, which is responsible for the weight-loss property of *Garcinia cambogia* [5]. A polyisoprenylated phenol derivative, garcinol is a potential therapeutic molecule and has been proven for its anti-oxidative, anti-inflammatory, and anti-cancer activities [6]. Garcinol has antioxidant activity equivalent to ascorbic acid, which has been evaluated and proven by 1,1-diphenyl-2- picrylhydrazyl (DPPH) method [4]. Garcinol was found to be structurally similar to curcumin (an anticancer agent), because of the presence of β-diketone and showed better anticancer activity compared to curcumin [7]. Garcinol reduces obesity by acting on the AMPK-ER stress axis [8] and was also reported to modulate gut microbiota and reduce obesity in mice [9]. To further support these preclinical studies and to accelerate the development of garcinol as a potential drug candidate, additional studies like in-vitro transport and metabolism are required [6].

There is a strong perception in the public that herbal products and herbal supplements are safe; however, herbal products also possess some adverse effects similar to synthetic drugs [10,11]. The main reasons for the occurrence of herb-drug interactions are the altered transport and metabolism of one drug by the other when they are administered together. CYP 450 enzymes are important drug metabolizing enzymes, which aid in the metabolism of most of the drugs. Many drug transporters have been identified in humans among which P-gp is the best characterized one. In intestinal enterocytes, P-gp actively secretes absorbed drugs back to the intestinal lumen, so inhibition or induction of P-gp by herbal products may results in the elevation and reduction in the drug concentration in the plasma [12]. These CYP enzymes and P-gp comprises an efficient bioavailability barrier for many orally administered drugs [13,14,15]. As most of the synthetic drugs and active ingredients of herbal drugs are the substrates of these CYPs drug metabolizing enzymes and P-gp (efflux transporter), ‘transport-metabolism interplay’ has gained much importance and attention [16,17].

Preliminary in vitro studies in our lab showed inhibitory effect of *Garcinia cambogia*, *Garcinia indica* extracts and formulations (studied along with pure garcinol) on CYP2B6 and CYP3A4. The detailed procedure and results are shown in Supplementary Material (Table S1). Based on the preliminary results, we conducted the present study of garcinol. The objective of the study is to evaluate the potential inhibition capability of garcinol on the main drug metabolizing enzyme isoforms i.e., on CYP1A2, CYP2C9, CYP2C19, CYP2B6, CYP2D6, CYP2E1, and CYP3A4 and on efflux transporter P-glycoprotein (P-gp). To the best of our knowledge, there are no reported studies on inhibition potential of garcinol and this would be the first study to report CYP 450 inhibition and P-gp inhibition potential of garcinol.

## 2. Materials and Methods

### 2.1. Chemicals and Reagents

Garcinol (CAS #78824-30-3), 1-hydroxy tacrine, hydroxybupropion 4-hydroxy diclofenac, 6-hydroxy paclitaxel, 4-hydroxy mephenytoin, 1-hydroxy midazolam, 6-hydroxychlorzoxazone, and dextrorphan were procured from Cayman Chemical Company (Ann Arbor, MI, USA). Rat liver microsomes were procured from BioreclamationIVT (Baltimore, MD, USA). Tacrine, bupropion, S-mephenytoin, diclofenac, paclitaxel, dextromethorphan, chlorzoxazone, and midazolam, dimethylsulfoxide (DMSO), phosphate buffered saline (PBS), and nicotinamide adenine dinucleotide phosphate reduced tetrasodium salt hydrate (NADPH) were purchased from Sigma-Aldrich (St. Louis, MO, USA). Formic acid and acetonitrile (HPLC grade) (>98% purified) were procured from Merck Specialities Pvt. Ltd. (Mumbai, India). Millipore water (in-house) was used for the entire study.

### 2.2. Animals

Healthy male Sprague-Dawley rats weighing about 200–220 g were purchased from Palamur Biosciences (Hyderabad, India) and were kept in a room which was environmentally controlled and maintained at 22–24 °C with a light/dark cycle of 12/12 h and 55 ± 5% relative humidity. All the animal protocols were reviewed and approved by (NIP/01/2019/PA/170) Institutional Animal Ethics Committee (IAEC) of the National Institute of Pharmaceutical Education and Research (NIPER), Hyderabad, India, and confined to the ‘Principles of Laboratory Animal Care’ (NIH Publication No. 85–23, revised 1985). Animals were acclimatized for one week before experimentation without restriction for food and water.

### 2.3. In-Vitro CYP 450 Incubation Studies

The activity of Garcinol on the eight CYP enzymes was characterized depending on their probe substrate reactions: CYP1A2 (Tacrine 1-hydroxylation), CYP2B6 (Bupropion hydroxylation), CYP2C9 (Diclofenac 4-hydroxylation), CYP2C19 (S-mephenytoin 4-hydroxylation), CYP2C8 (Paclitaxel 6α-hydroxylation), CYP2D6 (Dextromethorphano-demethylation), CYP2E1 (Chlorzoxazone 6-hydroxylation), and CYP3A4 (Midazolam1-hydroxylation). The pooled substrate approach has been validated and it is proven that there are no substrate-substrate interactions. Tacrine was used as CYP1A2 probe substrate instead of Phenacetin to avoid the possible interactions of Phenacetin with CYP2E1 probe substrate Chlorzoxazone [18]. The incubation mixture contained microsomal protein at the concentration of 0.5 mg/mL, 100 mM phosphate buffered saline (pH 7.4), nicotinamide adenine dinucleotide phosphate (NADPH), respective probe substrates at the concentrations equal to their respective K_m_ values and garcinol at different concentrations (30.0, 10.0, 3.0, 1.0, 0.33, 0.11 µM). The above incubation mixture was pre-incubated for 5 min at 37 °C and the reaction was started by adding NADPH and terminated by the addition of 200 µL of ice-cold acetonitrile containing internal standard (IS). Then the mixture was vortex mixed and centrifuged at 4000 rpm for 10 min at 4 °C and supernatant was separated and analysed using LC-MS/MS.

### 2.4. Time-Dependent Inhibitory Effects of Garcinol on CYP Isoforms Using IC_50_ Shift Assay Method

Garcinol was pre-incubated with rat liver microsomes to determine the time-dependent inhibition potential of garcinol on various CYP isoforms individually. After 30 min of incubation with and without NADPH separately, metabolites of each CYP probe substrates were quantified using LC-MS/MS. IC_50_ values with and without NADPH pre-incubation were calculated using graph-pad prism and change in IC_50_ fold was calculated to evaluate the potential of garcinol to inhibit the CYP enzymes irreversibly. If the curve shifts to lower IC_50_ value after the pre- incubation in presence of NADPH, it is said to be a time-dependent inhibition. Concentrations of the test compound and other procedure were same as reversible inhibition study mentioned under Section 2.3.

### 2.5. Characterization of Molecular Structures of Small Molecule and Protein Crystal Structures

Based on the CYP inhibition results, molecular docking analysis was performed for garcinol with all the CYP isozymes tested in-vitro using GLIDE module of Schrodinger software. The crystal structures of the human CYP1A2 (PDB ID: 2HI4), CYP2B6 (PDB ID: 5UFG), CYP2C8 (PDB ID: 2VN0), CYP2C9 (PDB ID: 4NZ2), CYP2C19 (PDB ID: 4GQS), CYP2D6 (PDB ID: 3QM4), CYP2E1 (PDB ID: 3GPH) [19] and CYP3A4 (PDB ID: 4K9W) [20] were used for molecular docking studies. Standard precision mode was selected for this ligand docking and the ligand was docked against the active sites of targeted proteins.

### 2.6. In-Vivo CYP 450 Studies

In-vivo CYP inhibition studies were designed to investigate the inhibition potential of garcinol on the drug metabolizing enzymes. Out of eight isoforms used in the in-vitro and modelling studies, two CYP isoforms were selected based on the in-vitro IC_50_ values i.e., CYP2B6 and CYP3A4 and were studied further. The rats were randomly divided into two groups as follows. Group I vehicle treatment group (*n* = 6), group 2 administered with garcinol (25 mg/kg) (*n* = 6) for 7 days. Garcinol was suspended in 0.5% sodium carboxy methyl cellulose (Na-CMC) and administered orally using gastric gavage. After 7 days of continuous oral administration of Garcinol (Group II), on the 8th day, both the groups (I & II) were treated with probe substrate mixture (bupropion; 10 mg/kg and midazolam; 10 mg/kg). Approximately 0.20 mL of blood samples were collected via retro- orbital route in tubes containing K_2_EDTA at pre-dosing (0 h), 0.15, 0.30, 1, 2, 4, 6, 8, and 24 h post-dosing. Blood samples were immediately centrifuged at 10,000 rpm for 5 min and plasma samples were collected and stored at −80 °C until LC-MS/MS analysis.

### 2.7. Analysis of CYP Metabolites

Metabolites of eight CYP450 selective substrates were analyzed using Sciex API4000 and Sciex 5500 Qtrap (negative mode) triple quadrupole mass spectrometers with Turbo-ion-spray (AB Sciex, Toronto, ON, Canada) in the positive ionization (ESI) mode (negative mode for 6-hydroxyl chlorzoxazone of CYP2E1), which was hyphenated to a binary 1200 RRLC (Agilent Technologies, Inc., Santa Clara, CA, USA) and HTS-PAL auto-sampler (CTC Analytics AG, Industriestrasse, Switzerland). An aliquot of 10 µL from each sample was injected onto Phenomenex kinetex C18 column (50 × 4.6 mm, 5 µm), and flow rate was 1.0 mL/min to the ionization source (split ratio was 1:1). The mobile phase composition was 0.1% formic acid in water as an aqueous phase (A) and 100% acetonitrile as an organic modifier (B) for positive mode and 0.1% formic acid in water as aqueous component (A) and 50% acetonitrile: 50% methanol as the organic modifier (B) for negative mode. A gradient LC method (time (min)/%B = 0.01/10, 0.80/90, 2.20/90, 2.40/10, 3.00/10) with a run time of 3.0 min was developed for the analysis of metabolites in positive mode, whereas a LC gradient method (time (min)/%B = 0.01/15, 0.08/85, 1.80/85, 1.81/15, 2.50/15) with a runtime of 2.5 min for analysing 6-hydroxyl chlorzoxazone (negative mode) in microsomal samples. The column and auto sampler were maintained at 40 °C and 4 °C, respectively. Concentrations of selective CYP-probe substrates used in this study and their respective metabolites are listed in Table 1. Peak areas were integrated for all components using Analyst Software Version 1.5.1. Eight chromatograms of metabolites were identified and characterized in Figure S1.

### 2.8. Western Blotting Analysis

For Western blot analysis, twelve rats were divided into two groups. The first group served as the control group receiving only the vehicle 0.5% sodium carboxy methyl cellulose (Na-CMC), and the second group rats were given an intragastrical dose of garcinol once a day (25 mg/kg suspended in 0.5% sodium carboxy methyl cellulose (Na-CMC)) for 10 days continuously. On the 10th day, all the rats were sacrificed under CO_2_ euthanasia. Brain and intestine were isolated immediately and stored at −80 °C until use. 10 mg of intestinal and brain samples were lysed using ice-cold radio immunoprecipitation assay lysis buffer containing 0.02 mM phenylmethane sulfonyl fluoride for 30 min and ultrasonicated for 60 s at 20-s intervals in an ice bath. These samples were transferred to centrifugation tubes and centrifuged for 10 min at 8000 g by maintaining temperature at 4 °C. Supernatants were estimated for protein concentration using BCA protein assay kit by following the manufacturer’s instructions. Protein denaturation was performed by reconstituting the supernatant in lammelle buffer and boiled for 5 mins at 97 °C. The denatured samples were later separated on 10% SDS-polyacrylamide gel electrophoresis and then transferred onto a nitrocellulose membrane. The membrane was then blocked with 3% bovine serum albumin in Tris-buffered saline-Tween 20 (TBS-T) for 1 hr at 37 °C. These immunoblots were probed with primary monoclonal antibody P-gp (1:1000) or β-actin (1:1000) overnight at 4 °C. Then the membranes were washed and incubated with the secondary antibody P-gp rabbit mAb (1:5000) for 1 h at 37 °C and washed with TBS-T for three times. The band intensity of P-gp protein was normalized to that of β-actin detected using Azure biosystems.

### 2.9. In-Vivo P-gp Pharmacokinetic Studies

Effect of single dose garcinol on the P-gp was evaluated in-vivo, 18 rats were divided into three groups (Group I- Group III). Group I (Control group): rats were administered intragastrically with a single dose of vehicle (0.5% CMC-Na), and after 2 h of vehicle administration, digoxin (0.5 mg/kg) was given to the rats by intragastric administration. Group II: Rats received a single dose of garcinol (25 mg/kg) and digoxin (0.5 mg/kg) after 2 h. Group III: Verapamil (potent P-gp inhibitor) was administered to these rats followed by the administration of digoxin (results not shown). 0.2 mL of blood was collected at 0, 0.25, 0.5, 1, 2, 4, 6, 8, 24, and 48 h, plasma was separated immediately by centrifuging blood samples at 5000 rpm for 5 min and stored at −80 °C until analysis.

### 2.10. Analysis of Digoxin in Rat Plasma

50 µL of plasma samples was crashed with ice-cold ACN containing internal standard (75 ng/mL) and vortexed gently and centrifuged at 10,000 rpm for 5 min. Supernatant was collected and submitted for LC-MS/MS analysis. LC-MS analysis was performed on LC-QTOF/MS (Agilent, 6540) and chromatographic separation was achieved on Waters X-Bridge C18 column (3.5 µm, 50 mm × 1.76 mm I.D) at 30 °C. Digoxin was analysed by a mobile phase consisting of solvent A (0.1% Formic acid in water) and solvent B (100% ACN) flow at a rate of 0.4 mL/min with a gradient program of 5 min. The program consisted of initial10 %B from 0.01 to 3.00 min, followed by 90% B from 3.01 to 4.00 min and again to 10% B from 4.01 to 5.00 min. [M+H]+ ions were identified and quantified for Digoxin and IS as 781.4369 and 515.2442, respectively. Optimized MS operating parameters were as follows: drying gas 10 L/min, gas temperature 325 °C, nebulizer at 40 psig, fragmentor voltage of 140 V and skimmer at 65 V.

### 2.11. Data Analysis

IC_50_ values were determined by comparing each CYP isozyme activity in the presence of different concentrations of inhibitor (Garcinol) with the control incubations containing no inhibitor (Negative control). A plot of the logarithm of inhibitor concentration versus percentage of the activity remaining after inhibition was drawn from which IC_50_ values were calculated by nonlinear least-square regression analysis using graph-pad prism software (ver. 4.03).

Pharmacokinetic parameters were calculated from the plasma concentration versus time curves of bupropion, hydroxyl bupropion, midazolam, and hydroxy midazolam obtained from each individual rat using a non-compartmental analysis using WinNonlin software. The parameters calculated directly from experimental data were total area under the plasma-concentration-time curve from zero to infinity (AUC_0-∞_) or the last measured time (AUC_last_) and terminal half-life (t_1/2_), the peak plasma concentration (C_max_) and time to reach peak plasma concentration (T_max_).

### 2.12. Statistical Analysis

All the results are presented as mean ± standard deviation (SD). The statistically significant differences of the results were analyzed using one-way ANOVA and are considered to be statistically significant at a level of *p* value < 0.05.

## 3. Results

### 3.1. Screening for Reversible and Irreversible Inhibition of Garcinol on the Eight CYP Isoforms

**Figure 1:**
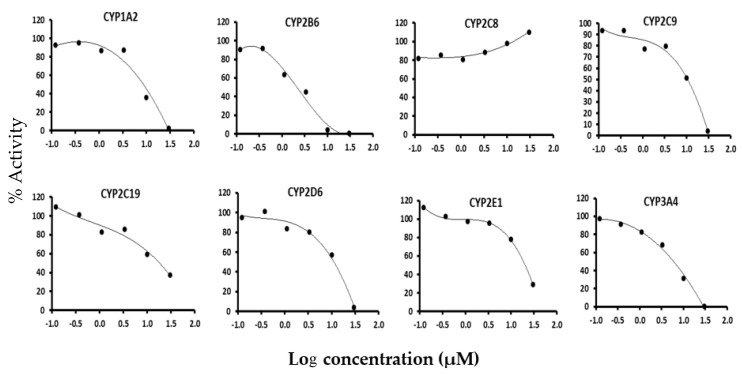
IC_50_ curves of garcinol for CYP450 activities using the cocktail substrate including CYP1A2 for tacrine 1-hydroxylase, CYP2B6 for bupropion hydroxylase, CYP2C8 for paclitaxel 6-hydroxylase, CYP2C9 for diclofenac 4-hydroxylase, CYP2C19 for S-mephenytoin 4-hydroxylase, CYP2D6 for dextromethorphan O-demethylase, CYP2E1 for chlorzoxazone 6-hydroxylase and CYP3A4 for midazolam 1′-hydroxylase.

Garcinol displayed strong inhibitory effects on CYP1A2, CYP2B6, CYP2C9, CYP2C19, CYP2D6, CYP2E1, and CYP3A4 with IC_50_ values of 7.6, 2.1, 8.0, 16.4, 9.5, 19.0, and 5.1 µM, respectively, and no apparent inhibition on CYP2C8 as shown in Figure 1. IC_50_ values of garcinol and the well-known inhibitors used for the eight CYP isoforms (CYP1A2, CYP2C9, CYP2C19, CYP2D6, CYP2E1, CYP2B6, CYP2C8, and CYP3A4) are reported in Table 2. In the time dependent inhibition study, no significant shift in the IC_50_ of any of the CYP isoform was observed.

### 3.2. Molecular Docking Studies of Garcinol on CYP2B6 and CYP3A4

Molecular docking and binding energy calculation results are summarized in Table S2. These results revealed that the docked garcinol showed comparable glide docking score to the reference known inhibitors and they interacted with active site amino acid residues of CYP3A4 and CYP2B6. Computationally it is worth mentioning that garcinol was well docked at the active sites of CYP3A4 and CYP2B6 but not to the other CYPs (Table S2). Thereby, it could be claimed that garcinol will be a promising inhibitor for CYP3A4 and CYP2B6 of CYPs family. The predicted binding modes and the detailed protein-inhibitor interactions of garcinol with CYP2B6 and CYP3A4 are illustrated in Figure S2A,B, respectively. From the molecular docking, it was observed that garcinol established three hydrogen bond interactions with the active residues Lys100, Asn117, and Asp385 of CYP2B6. Bridged-ring ketone of bicycle nonane moiety (acts as hydrogen bond acceptor) was involved in H-bond interaction with side chain amino group of Lys100 (d = 2.3 Å) and the keto group at the third position of bicycle nonane moiety (acts as hydrogen bond acceptor) was involved in H-bond interaction with side chain amino group of ASN117 (d = 2.9 Å). The parahydroxy phenyl moiety of garcinol (acts as hydrogen bond donor) was involved in H-bond interaction with back bone carboxylic group of Asp385 (d = 2.1 Å).

Additionally, a π-cation interaction (arene-cation interaction) by 3, 4-dihydroxyphenyl group of garcinol formed with the Lys100. Furthermore, several hydrophobic interactions were observed between the compound and the active site residues that stabilized the binding of the garcinol in the active site of CYP2B6 e.g., Ile101, Pro106, Phe108, Tyr111, Phe115, Ala116, and Ile370. Additionally, our molecular modeling studies also predicted that garcinol has reasonable affinity for CYP3A4. The binding model of the garcinol and CYP3A4 protein exposed that the compound showing two hydrogen bonding interactions with the key residues of the active site of CYP3A4 (Ile369, Arg372) and also having comparable hydrophobic interactions with Phe57, Phe108, Met114, Ile120, Leu210, Leu211, Phe213, Phe241, Ile300, Ile301, Phe304, Ala305, Ile369, Ala370, Met371, Leu482, and Leu483. From this modeling study we observed that garcinol was showing more affinity towards CYP2B6 and also observed reasonable affinity towards CYP3A4. These molecular modeling studies suggest that the inhibitory activity of garcinol was established by occupying the active sites of CYP2B6 and CYP3A4 and by making favorable interactions with its key residues.

### 3.3. Effects of Garcinol on the Activity of CYP Isoforms In-Vivo

**Figure 2:**
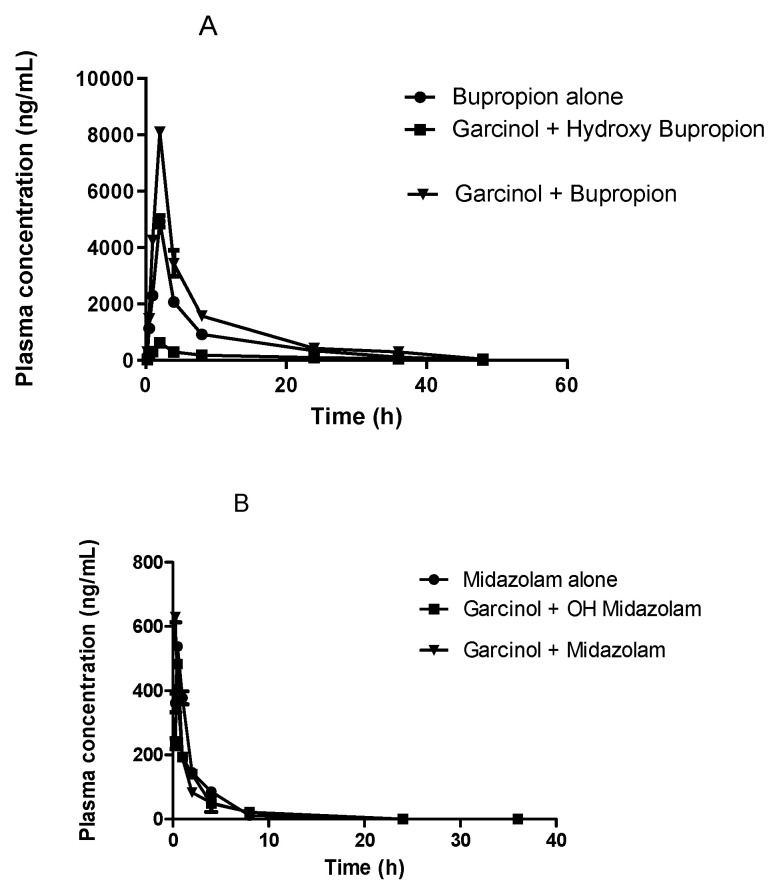
Mean plasma concentrations of bupropion (**A**) and midazolam (**B**) after oral administration of garcinol at a dose of 25 mg/kg in rats.

The plasma concentrations of metabolites hydroxy bupropion (CYP2B6) and 1-hydroxy midazolam (CYP3A4) in the absence and presence of garcinol were calculated and the pharmacokinetic parameters are shown in Table 3. These results indicated that there was an increase in the metabolites formed in the rats that had not received garcinol, compared to the group which received garcinol. The AUC_0-t_ and C_max_ of metabolites after co-administration with garcinol were comparable to those without garcinol and there was a significant change in the pharmacokinetic parameters of substrates and their metabolites formed with or without the administration of garcinol, demonstrating that the pharmacokinetics of bupropion and midazolam were affected by the presence of garcinol, which has potential to inhibit CYP enzymes. Plasma concentration vs. time plots were drawn using graph pad prism and are shown in Figure 2.

### 3.4. Expression of P-gp by Garcinol in Rat Brain and Intestinal Tissues

**Figure 3:**
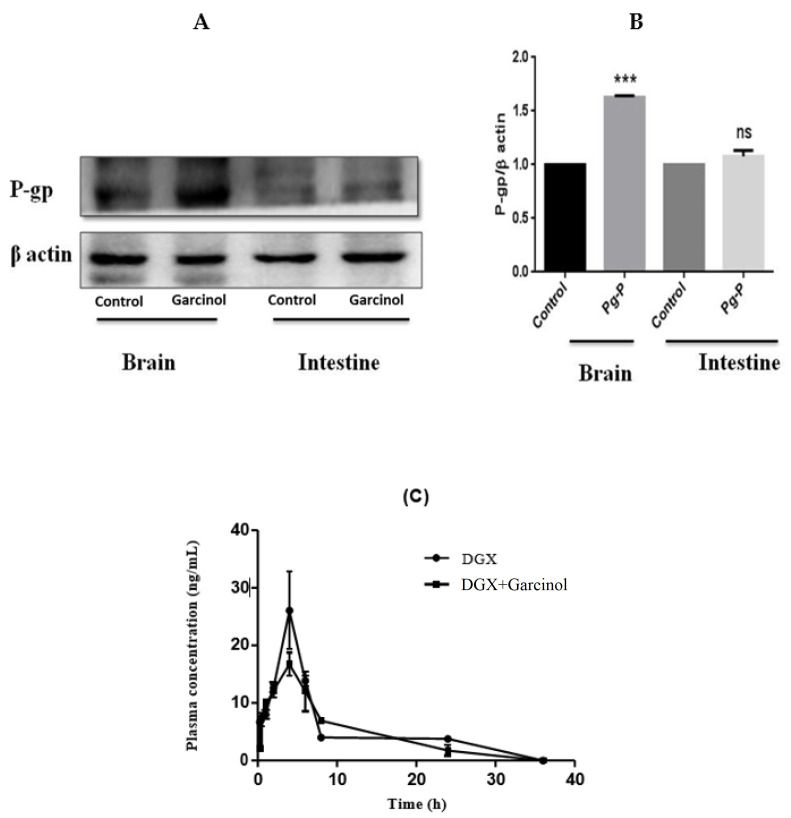
The effect of garcinol on the P-gp expression in brain and intestinal tissues (**A**) and graph showing the difference between control and garcinol treated groups (*** *p* < 0.05) (**B**) and plasma concentration vs. time graph showing the effect of garcinol on the P-gp function in SD rats (**C**).

Rat brain and intestinal tissue homogenates were used to evaluate the potential of garcinol to alter the expression of P-gp in-vivo. There was a significant difference in the expression in brain and intestinal tissues between two groups, as shown in Figure 3A. P-gp expression was increased by 1.62 and 1.07 fold, respectively, in the brain and intestinal tissues between the control group and the garcinol-treated group (*p* < 0.05), as shown in Figure 3B.

### 3.5. Effect of Single Dose of Garcinol on Plasma Concentration of P-gp Substrate in SD Rats

Effect of garcinol on the pharmacokinetics of digoxin was investigated in rat plasma. The plasma concentration-time profiles of digoxin administered singly or along with garcinol are shown in Figure 3C and pharmacokinetic parameters of these groups are mentioned in Table 4.

## 4. Discussion

Herbal medicine (HM) has been administered along with other drugs by many people without any awareness of interactions, which intensifies the occurrence of herb–drug interactions, especially in combination therapies. Though HM is naturally-occurring, the complex nature of various phytomolecules may interact with conventional drugs. The main interactions are related to the transport and metabolism of one drug in the presence of other drugs. CYP enzymes are involved in the metabolism of most drugs and P-gp is the efflux transporter present in the brain, intestine, kidney, and liver [21]. These enzymes and transporters are prone to induction, inhibition, and activation by herbal constituents [22]. To conclude, modulation of activity and expression of these enzymes by HMs has been responsible for several herb–drug interactions reported in the past. In the present study, inhibitory effects of garcinol on eight CYP enzymes and on P-gp transporter was evaluated in-vitro and in-vivo to understand the possibility of herb-drug interactions with concomitantly administered drugs.

The CYP inhibition potential of garcinol on eight CYP isoforms was evaluated by cocktail method. We observed that garcinol showed inhibition towards CYP1A2, CYP2B6, CYP2C9, CYP2C19, CYP2D6, CYP2E1, and CYP3A4 with IC_50_ values 7.6, 2.1, 8.0, 16.4, 9.5, 19, and 5.1 µM, respectively and CYP2C8 was negligibly inhibited, as mentioned in results. Garcinol also exhibited inhibition of CYP2B6 and CYP3A4 in the rat plasma after oral administration. However, in a pre-incubation study using rat liver microsomes and NADPH, garcinol did not show shift of inhibition against tested CYP isoforms. In order, to assess the binding of garcinol towards different CYP isoforms, we performed molecular docking studies of garcinol and the respective known inhibitors at the active pocket of different CYP isoforms. We observed that garcinol was well lodged at the binding site of CYP3A4 and CYP2B6 with the binding energy of −34.634 and −32.432 Kcal/mol, respectively. This further confirmed the inhibitory potential of garcinol.

In addition to the CYP inhibition studies, down-regulation and inhibition potential of garcinol towards P-gp transporter was evaluated. From western blotting study, it was observed that garcinol increased the expression of P-gp in brain and intestinal tissues. Effects of garcinol on the pharmacokinetics of P-gp substrate was investigated in-vivo with and without co- administration of garcinol. Digoxin, a P-gp substrate, was administered to rats orally. Intragastric co-administration of garcinol resulted in increase in the AUC of digoxin. In addition to showing direct inhibition or induction of P-gp, many P-gp modifiers also alters the expression of P-gp [23]. In this study, Western blotting results directly confirmed that P-gp expression was significantly increased in brain tissue, while it increased to a moderate extent in intestinal tissue. These results suggest that garcinol is not an inhibitor of P-gp; instead, it increases the activity of P-gp, thereby decreasing the bioavailability of P-gp substrates in rats.

## 5. Conclusions

The results of this study depict the CYP inhibition potential of garcinol, thus admonishing co-administration of CYP substrates with garcinol for various therapies. Also, garcinol did not inhibit P-gp, thus decreasing the bioavailability of P-gp substrates for its therapeutic activity. Further clinical studies are required to fully assess the safety of garcinol in terms of CYP2B6 and CYP3A4 inhibition.

## Figures and Tables

**Table 1:** Substrates, their metabolites, and their LC-MS/MS conditions of the reaction for CYPs using cocktail assays.

CYP Isoform	Substrates	Concentration (µM)	Metabolites	Transition (m/z)	Polarity	CE (eV)
CYP1A2	Tacrine	50	1-Hydroxy Tacrine	215.3 → 182.1	ES^+^	43
CYP2B6	Bupropion	50	Hydroxy Bupropion	256.1 → 238.0	ES^+^	7
CYP2C8	Paclitaxel	50	6-Hydroxy Paclitaxel	870.0 → 286.0	ES^+^	17
CYP2C9	Diclofenac	50	4-Hydroxy Diclofenac	312.2 → 230.2	ES^+^	12
CYP2C19	S-Mephenytoin	300	4-Hydroxy Mephenytoin	235.2 → 150.2	ES^+^	25
CYP2D6	Dextromethorphan	50	Dextrorphan	258.2 → 157	ES^+^	25
CYP2E1	Chlorzoxazone	2000	6-Hydroxy Chlorzoxazone	183.0 → 120.0	ES^-^	−28
CYP3A4	Midazolam	20	1-Hydroxy Midazolam	342.2 → 203.0	ES^+^	18
Internal Standard	-	-	Telmisartan	515.4 → 276.1	ES^+^	50

ES^+^: Electrospray positive ion mode, ES^-^: Electrospray negative ion mode, CE: Collision energy.

**Table 2:** Inhibition parameters of garcinol towards eight CYPs in rat liver microsomes.

CYP Isoform	Substrates	Inhibitors	IC_50_ (µM) Garcinol
CYP1A2	Tacrine	α-naphthoflavone	7.6 *
CYP2B6	Bupropion	Ticlopidine	2.1 *
CYP2C8	Paclitaxel	Quercetin	>30
CYP2C9	Diclofenac	Sulphaphenazole	8.0 *
CYP2C19	S-Mephenytoin	N-Benzyl nirvanol	16.4 #
CYP2D6	Dextromethorphan	Quinidine	9.5 *
CYP2E1	Chlorzoxazone	4-Methyl Pyrazole	19 #
CYP3A4	Midazolam	Ketoconazole	5.1 *

* Significant inhibition, # moderate inhibition.

**Table 3:** Mean (±standard deviations) pharmacokinetic parameters of bupropion, hydroxy bupropion, midazolam, and hydroxy midazolam alone in rats with or without oral administration of garcinol.

Parameters	Control Group (Bupropion Alone)	Bupropion with Garcinol	Hydroxy Bupropion with Garcinol
C_max_ (ng/mL)	4929.05 ±198.13	8095.72 ± 120.63	632.02 ± 30.55
AUC_0-∞_ (h*ng/mL)	29,335.73 ± 2034.86	48,768.38 ±1279.63	6200.97 ± 106.97
AUC_last_ (h*ng/mL)	29,212.34 ± 2057.52	48,245.49 ± 1243.61	6057.86 ± 101.41
T_max_ (h)	1.5 ± 2.31	2.5 ± 3.52	1 ± 1.93
t_1/2_ (h)	6.11 ± 1.03	7.93 ± 0.08	10.23 ± 0.04
Cl (ml/h/kg)	341.99 ± 24.16	205.14 ± 5.31	NA
C_max_ (ng/mL)	537.63 ± 10.24	828.69 ± 15.65	482.85 ± 14.34
AUC_0-∞_ (h*ng/mL)	1023.21 ± 58.29	1138.22 ± 77.38	607.65 ± 34.26
AUC_last_ (h*ng/mL)	998.54 ± 53.44	1237.81 ± 18.59	737.45 ± 66.95
T_max_ (h)	0.25 ±3.2	0.5 ±1.23	0.25 ± 1.92
t_1/2_ (h)	1.51 ± 0.18	3.12 ± 0.88	2.15 ± 0.36
Cl (ml/h/kg)	9795.11 ± 577.02	11,996.63 ± 1083.16	NA

NA—Not applicable.

**Table 4:** Mean (±standard deviations) pharmacokinetic parameters of digoxin (DGX) in SD rats with or without oral co-administration of garcinol.

Groups	C_max_ (ng/mL)	AUC_last_ (h*ng/mL)	AUC_0-∞_ (h*ng/mL)	t_1/2_ (h)	Cl (ml/h/kg)
DGX	26.075 ± 5.23	172.08 ± 12.26	254.06 ± 19.23	15.06 ± 0.35	984.06 ± 56.87
DGX + Garcinol	16.75 ± 4.22	151.39 ± 0.58	172.27 ± 12.58	7.318 ± 1.75	1474.76 ± 85.34

## Data Availability

Not applicable.

## Supplementary Materials

The following are available online at https://www.mdpi.com/article/10.3390/membranes11120992/s1, Figure S1: LC-MS/MS chromatograms of eight CYP-450 specific metabolites, Figure S2: A. (a) Docking model of garcinol at the active site of CYP2B6 (PDB ID: 5UFG) and (b) its ligand-protein interactions in the binding site of CYP2B6 (Purple: Protein, 2B6; Green: Ligand, Garcinol; Red: Hydrogen bonds); B. (a) Docking model of garcinol at the active site of CYP3A4 (PDB ID: 4K9W) and (b) its ligand-protein interactions in the binding site of CYP3A4 (Purple: Protein,2B6; Green: Ligand, Garcinol; Red: Hydrogen bonds), Table S1: Inhibition parameters of herbal slimming agents towards eight CYPs in rat liver microsomes, Table S2: GLIDE docking results for Garcinol at the active sites of different CYPs.
