# Supplementary material for: Cytochrome P450 and P-gp Mediated Herb-Drug Interactions and Molecular Docking Studies of Garcinol
Source: Membranes (Basel). 2021 Dec 19;11(12):992. doi: 10.3390/membranes11120992 (PMC8705362; doi:10.3390/membranes11120992)
Supplement: Supplementary file 1 [file membranes-11-00992-s001.zip › membranes-1500780-supplementary.pdf]

membranes

MDPI

Supplementary Materials

Cytochrome P450 and P-gp Mediated Herb-Drug Interactions and Molecular Docking Studies of Garcinol

Lavanya Bolla [1], Pratima Srivastava [1], Velayutham Ravichandiran [2,*] and Satheesh Kumar Nanjappan [2,*]

[1] Aragen Life Sciences Pvt. Ltd. (Formerly known as GVK Biosciences Pvt. Ltd.), IDA Nacharam, Hyderabad 500076, India; lavanyabolla225@gmail.com (L.B.); pratima.srivastava@gvkbio.com (P.S.)
[2] Department of Natural Products, National Institute of Pharmaceutical Education and Research (NIPER), Kolkata, Chunilal Bhawan 168, Maniktala Main Road, Kolkata 700054, India
* Correspondence: director@niperkolkata.edu.in (V.R.); satheesh.niperk@gmail.com (S.K.N.)

Table S1. Inhibition parameters of herbal slimming agents towards eight CYPs in rat liver microsomes.

CYP Isoform	Substrates	Inhibitors	IC50 (µM/µg/mL)				
			Garcinia cambogia Formulation	*Garcinia cambogia* Extract	*Garcinia indica* Formulation	*Garcinia indica* Extract	Garcinol
**CYP1A2**	Tacrine	α-naphthoflavone	>30	>30	>30	>30	7.6*
**CYP2B6**	Bupropion	Ticlopidine	>30 (48.3)*	>30 (62.3)#	>30 (52.6)#	>30 (75.5)#	2.1*
**CYP2C8**	Paclitaxel	Quercetin	>30	>30	>30	>30	>30
**CYP2C9**	Diclofenac	Sulphaphenazole	>30	>30	>30	>30	8.0*
**CYP2C19**	S-Mephenytoin	N-Benzyl nirvanol	>30	>30	>30	>30	16.4#
**CYP2D6**	Dextromethorphan	Quinidine	>30	>30	>30	>30	9.5*
**CYP2E1**	Chlorzoxazone	4- Methyl Pyrazole	>30	>30	>30	>30	19#
**CYP3A4**	Midazolam	Ketoconazole	>30 (84.7) #	>30 (75.2) #	>30 (93.5)#	>30 (89.2)#	5.1*

* Significant inhibition, # moderate inhibition; IC_{50} values mentioned in brackets are below 100 µg/mL but more than 30 µg/mL which is considered to show inhibition for Formulation and extracts.

Extraction

Garcinia cambogia, *Garcinia indica* extracts and formulations were accurately weighed and transferred to separate eppendorf tubes and dissolved in MQ Water to make 50 mg/mL stock solution. The tubes were first shaken vigorously and sonicated for 30 min and filtered using 0.45 µM syringe filter. These stock solutions were kept at -20 ^{0}C and used for further studies.

CYP Inhibition study

The entire procedure is same as mentioned in section 2.3 except for various concentrations of the formulations and extracts (0.41-100 µg/mL) and garcinol (0.11-30 µM) were used in the study.

Table S2. GLIDE docking results for Garcinol at the active sites of different CYPs.

S. no	CYPs	Ligand name	Docking score	Binding energy (kcal/mol)	Interactions			
					H- bonds	π- cat-ion	π- π	Hydrophobic
1	CYP1A2 (PDB ID: 2HI4)	Garcinol	-	-	-	-	-	-
		alpha-Naphthoflavone	-11.252	-62.439	-	-	Phe226	Ile117, Phe125, Phe226, Val227, Phe256, Phe260, Ala317, Leu382, Ile386, Leu497
2	CYP2B6 (PDB ID: 5UFG)	Garcinol	-3.016	-32.432	Lys100, Asn117, Asp385	Lys100	-	Ile101, Pro106, Phe108, Tyr111, Phe115, Ala116, Ile370
		Ticlopidine	-7.250	-25.191	-	Phe297	-	Ile101, Val104, Phe108, Val114, Phe115, Phe206, Ile209, Phe297, Ala298, Leu362, Leu363, Val367, Val477
3	CYP2C8 (PDB ID: 2VN0)	Garcinol	-	-	-	-	-	-
		Quercitin	-6.950	-26.853	Phe201, Asn202, Glu300	-	Phe205	Ile113, Phe201, Phe205, Leu208, Val296, Ala297, Val362, Val366, Ile476, Val477
4	CYP2C9 (PDB ID: 4NZ2)	Garcinol	-	-	-	-	-	-
		Sulphaphenazole	-6.139	-8.326	-	-	-	Val113, Phe114, Ile205, Leu208, Val292, Ala297, Leu361, Leu362, Leu366, Pro367, Ala477, Val479
5	CYP2C19 (PDB ID: 4GQS)	Garcinol	-	-	-	-	-	-
		Benzylnirvanol	-6.072	-1.294	-	-	-	Phe100, Leu102, Val113, Phe114, Leu201, Ile205, Val208, Leu233, Leu237, Ala297, Ile362, Leu366, Phe476
6		Garcinol	-	-	-	-	-	-

	CYP2D6 (PDB ID: 3QM4)	Quinidine	-7.506	-20.304	Ser304	-	-	Leu110, Phe120, Ala209, Leu213, Phe247, Ala305, Val308, Ile369, Val370, Phe483, Leu484
7	CYP2E1 (PDB ID: 3GPH)	Garcinol	-	-	-	-	-	-
		4-Methylpyrazole	-5.028	-17.042	-	-	-	Ile115, Phe207, Phe298, Ala299, Leu363, Val364, Leu368, Phe478
8	CYP3A4 (PDB ID: 4K9W)	Garcinol	-7.310	-34.634	Ile369, Arg372	-	-	Phe57, Phe108, Met114, Ile120, Leu210, Leu211, Phe213, Phe241, Ile300, Ile301, Phe304, Ala305, Ile369, Ala370, Met371, Leu482, Leu483
		Ketoconazole	-8.236	-49.138	Asp76, Thr309, Met371	Phe57	Arg106, Phe213	Ile50, Tyr53, Phe57, Phe108, Leu211, Phe213, Phe215, Phe304, Ala305, Ile369, Ala370, Met371, Leu482, Leu483

Note: Garcinol was found to have no interaction against other CYP isoforms (CYP1A2, CYP2C8, CYP2C9, CYP2C19, CYP2D6, and CYP2E1) therefore the values were not mentioned.

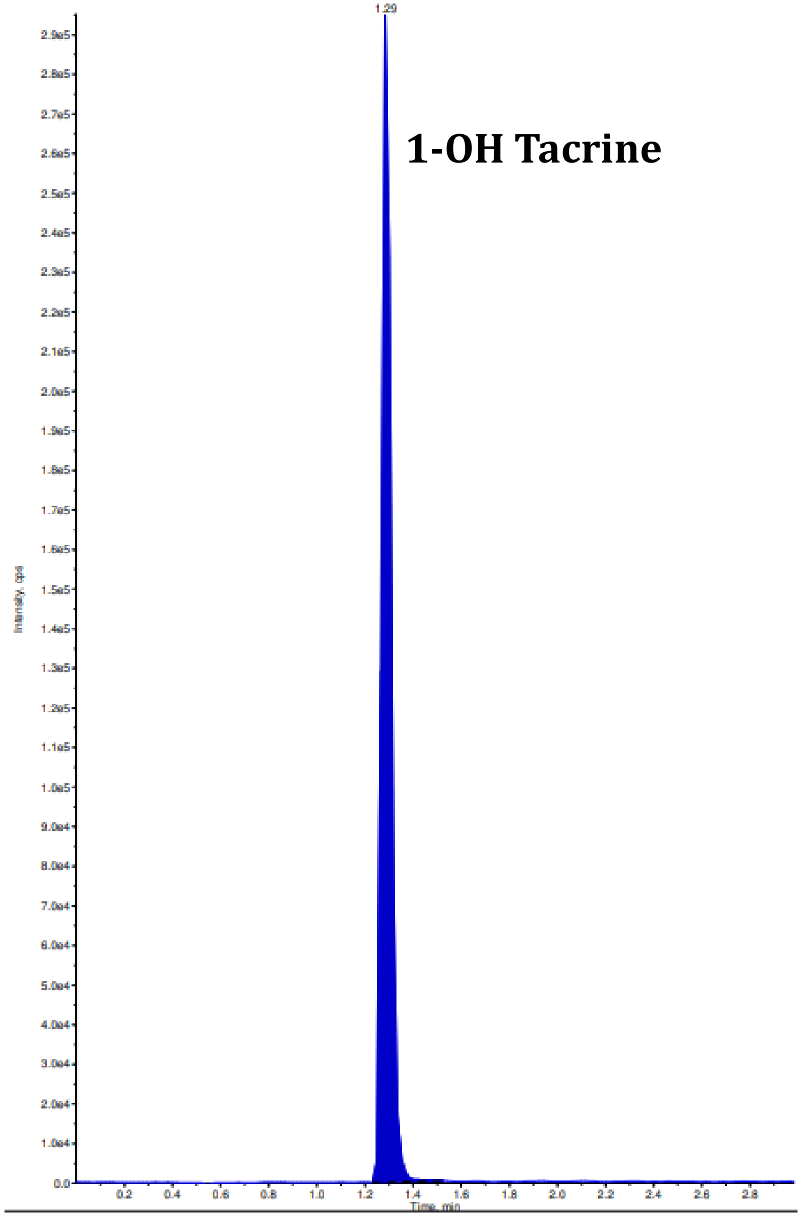
1.29
1-OH Tacrine
Intensity, cps
Time, min

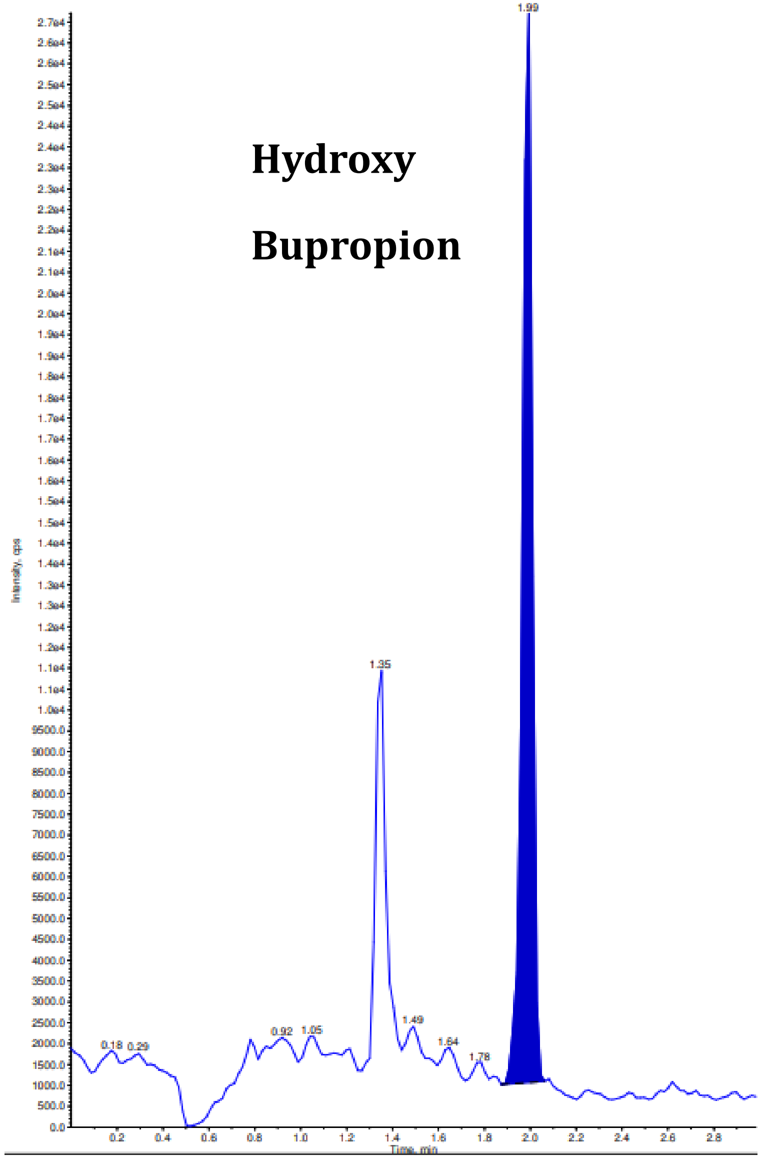
1.99
Hydroxy
Bupropion
1.35
Intensity, cps
Time, min

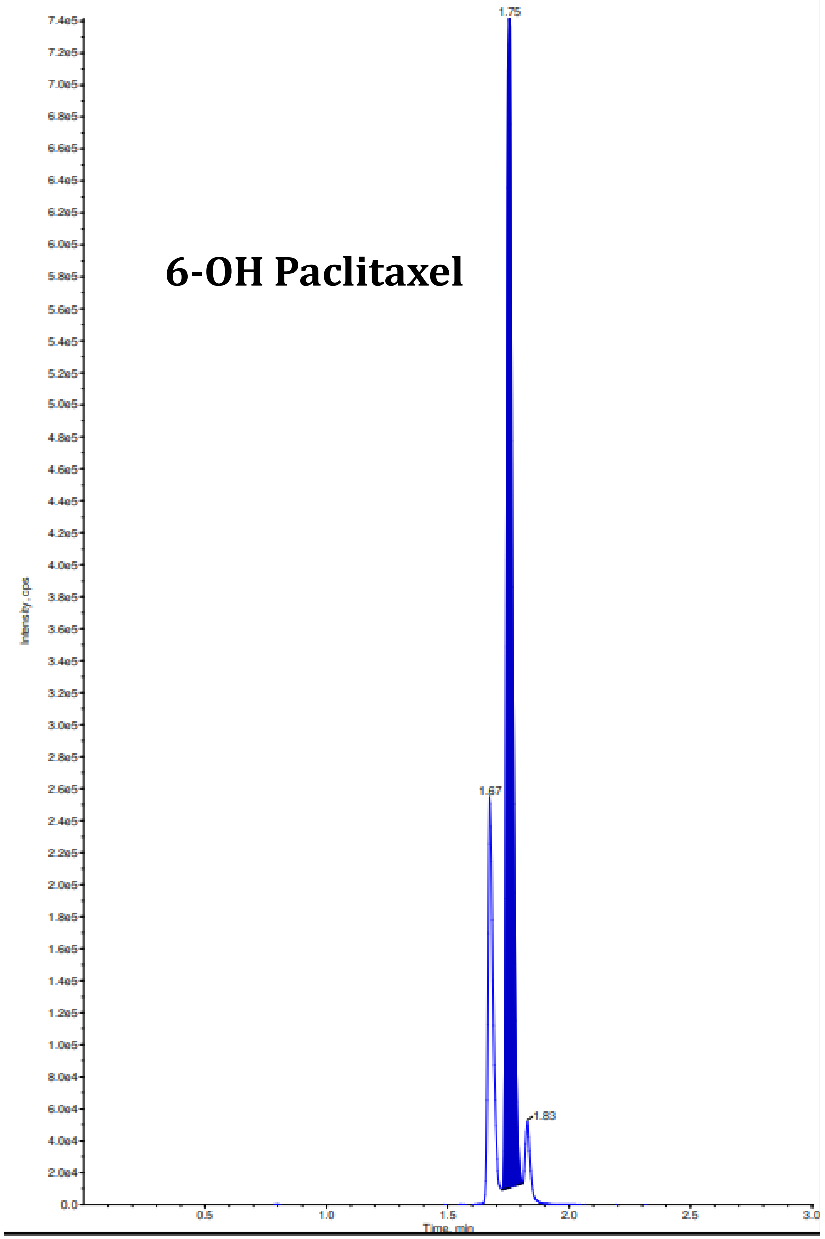
6-OH Paclitaxel
1.75
1.67
1.83
Intensity, cps
Time, min

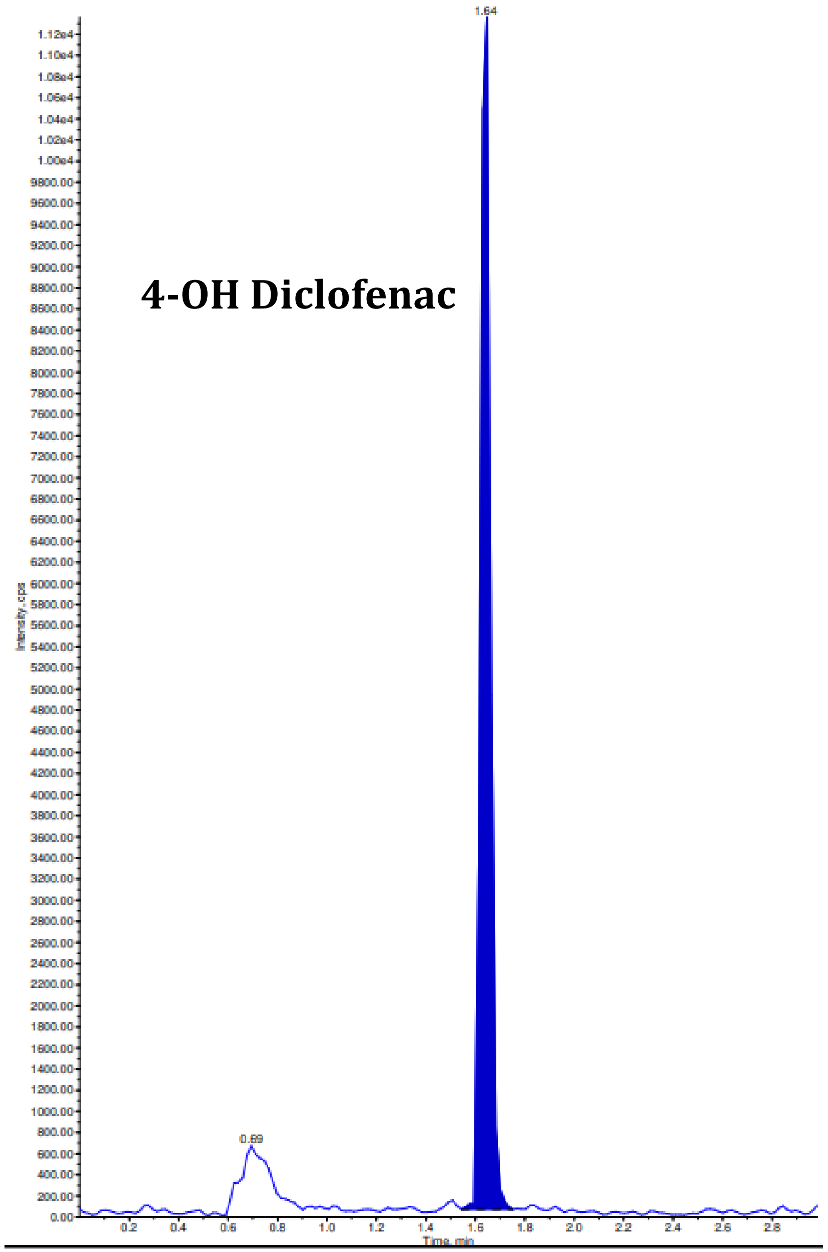
4-OH Diclofenac
1.64
0.69
Intensity, cps
Time, min

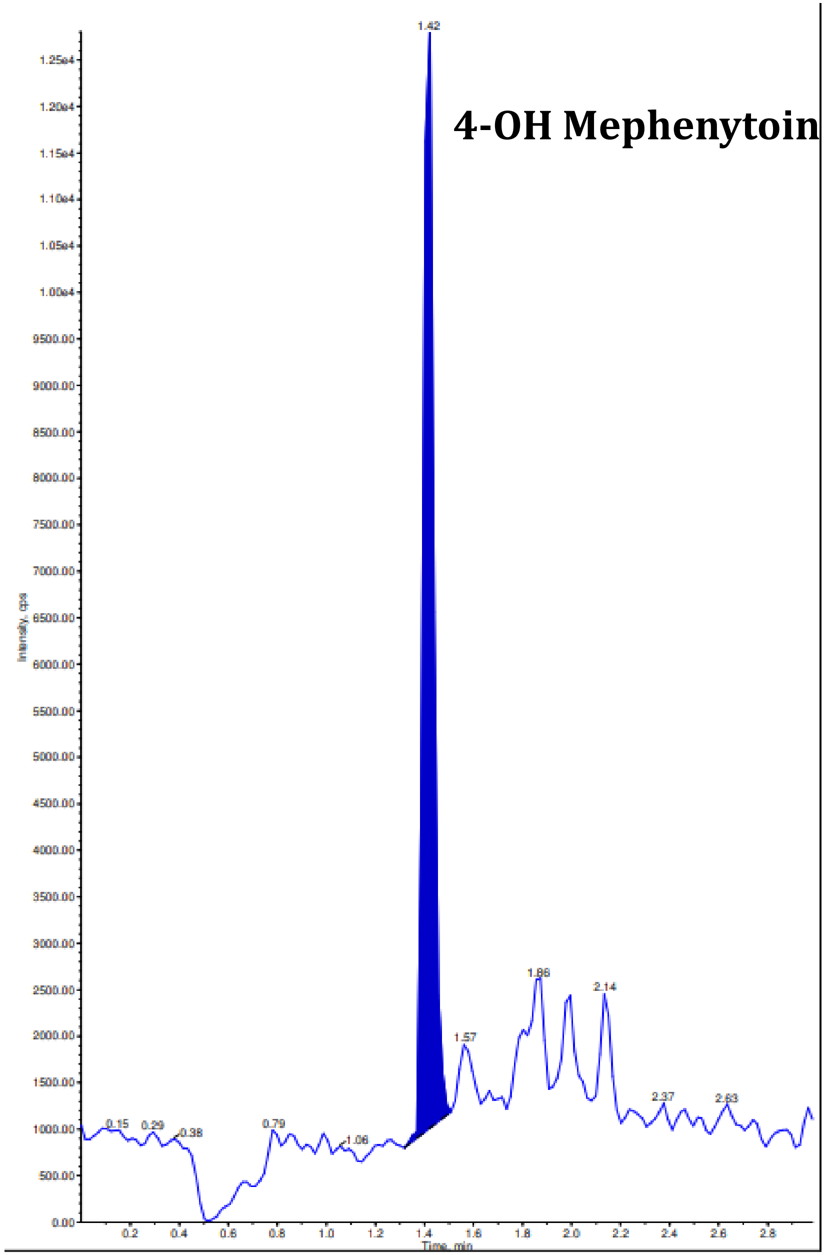
4-OH Mephenytoin
Intensity, cps
Time, min
1.42
0.15
0.29
0.38
0.79
1.06
1.57
1.86
2.14
2.37
2.63

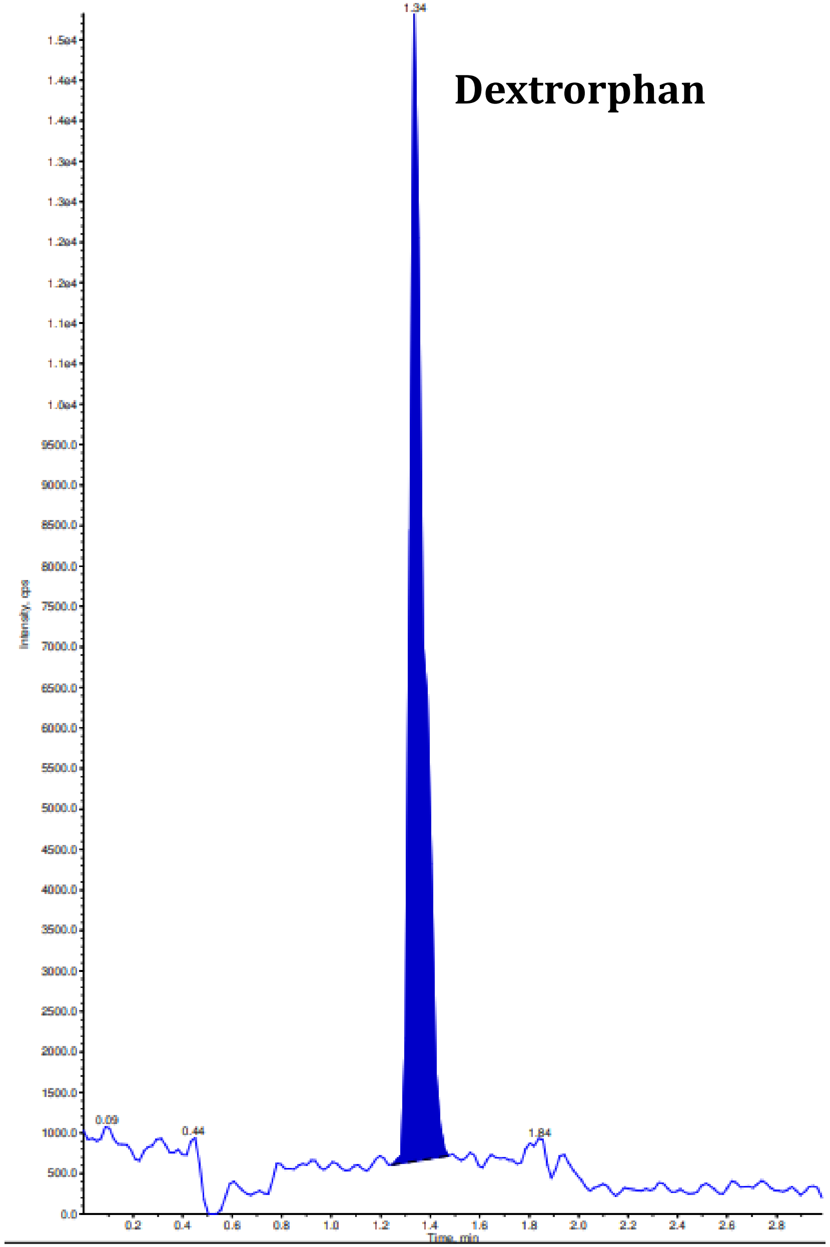
Dextrorphan
Intensity, cps
Time, min
1.34
0.09
0.44
1.84

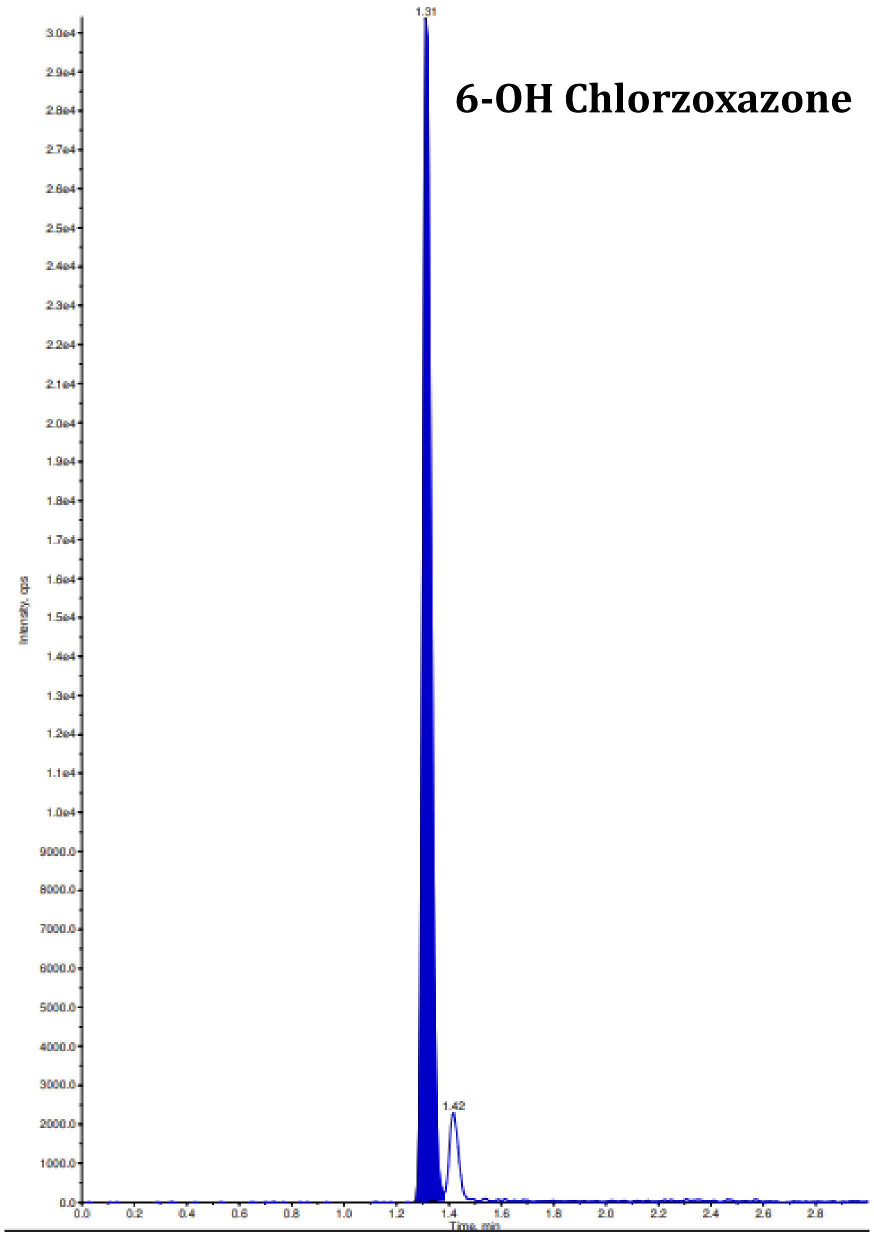
6-OH Chlorzoxazone
1.31
1.42
Intensity, cps
Time, min

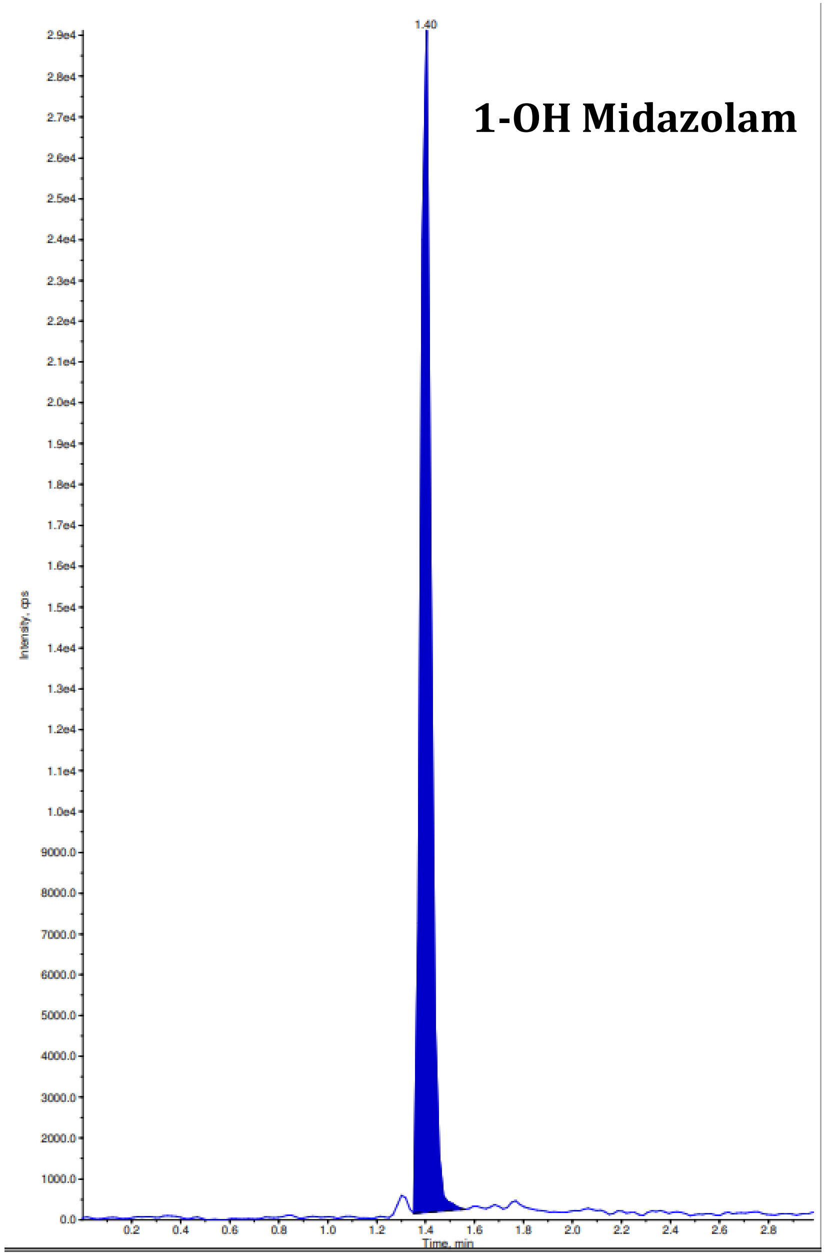
1-OH Midazolam
1.40
Intensity, cps
Time, min

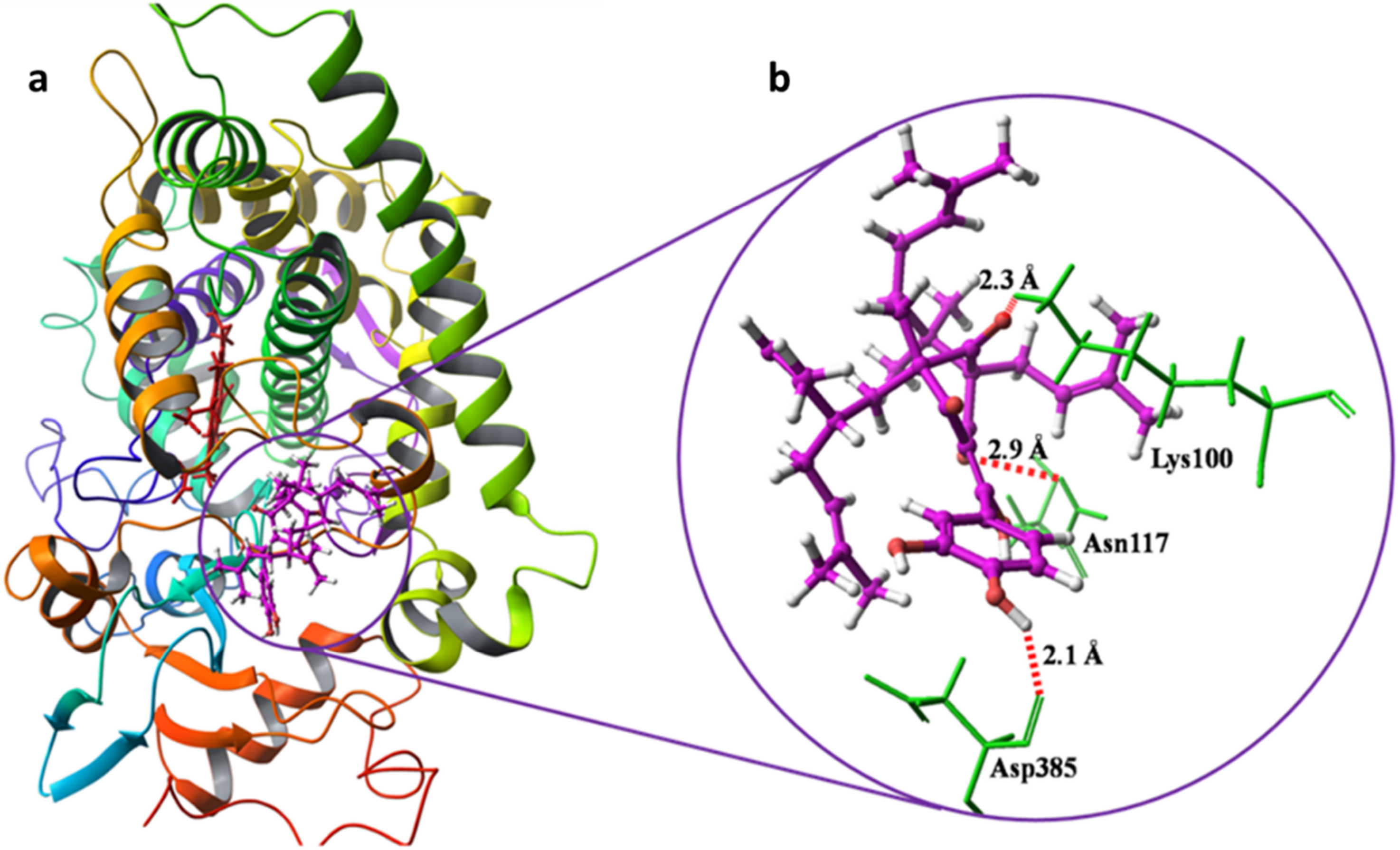

Figure S1. LC-MS/MS chromatograms of eight CYP-450 specific metabolites.

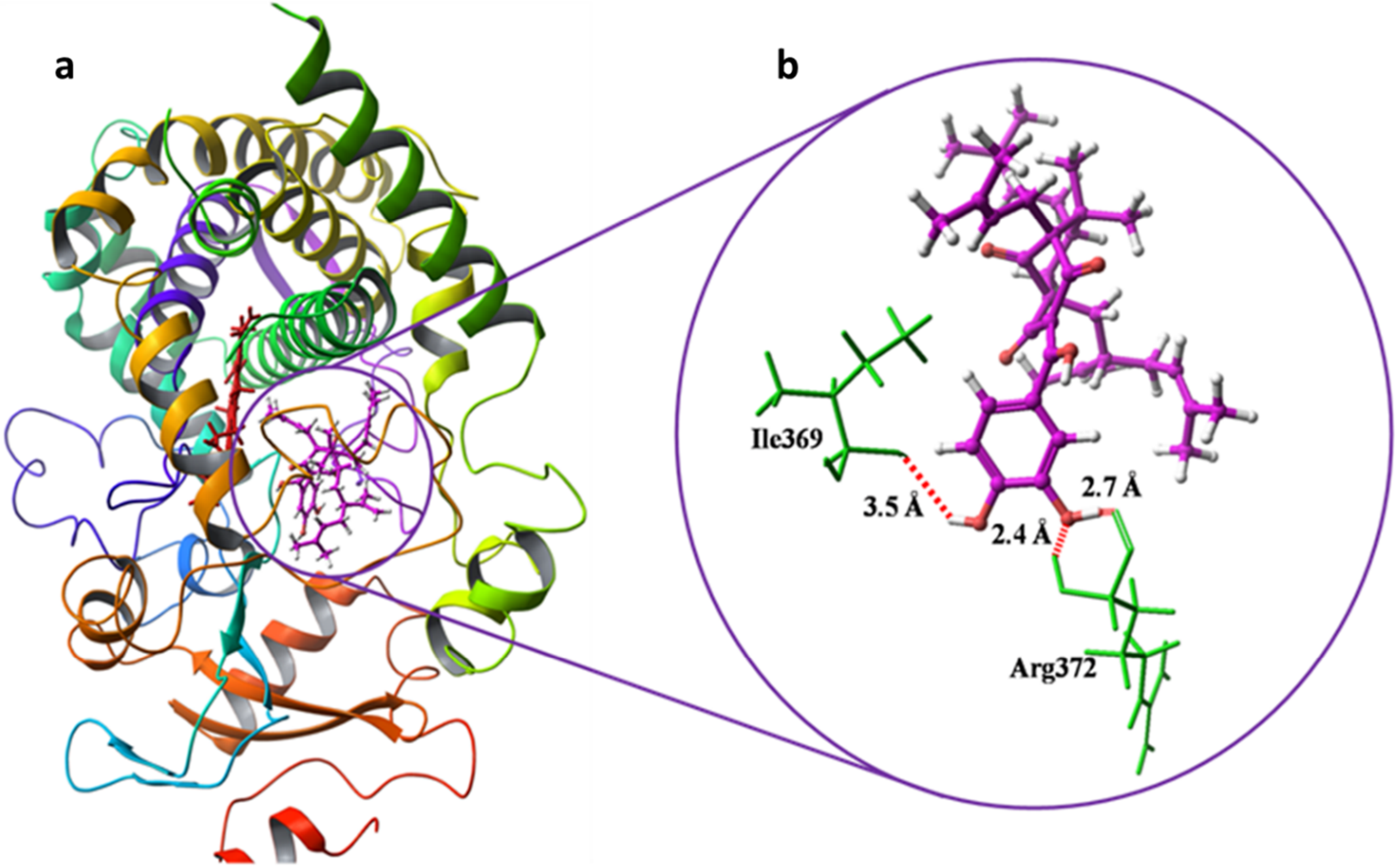

Figure S2. A. (a) Docking model of garcinol at the active site of CYP2B6 (PDB ID: 5UFG) and (b) its ligand-protein interactions in the binding site of CYP2B6 (Purple: Protein, 2B6; Green: Ligand, Garcinol; Red: Hydrogen bonds); B. (a) Docking model of garcinol at the active site of CYP3A4 (PDB ID: 4K9W) and (b) its ligand-protein interactions in the binding site of CYP3A4 (Purple: Protein,2B6; Green: Ligand, Garcinol; Red: Hydrogen bonds).
